# Tackling complexities in understanding the social determinants of health: the contribution of ethnographic research

**DOI:** 10.1186/1471-2458-11-S5-S6

**Published:** 2011-11-25

**Authors:** Mridula Bandyopadhyay

**Affiliations:** 1 Mother & Child Health Research, La Trobe University, 215 Franklin Street, Melbourne Victoria 3000, Australia

## Abstract

**Objective:**

The complexities inherent in understanding the social determinants of health are often not well-served by quantitative approaches. My aim is to show that well-designed and well-conducted ethnographic studies have an important contribution to make in this regard. Ethnographic research designs are a difficult but rigorous approach to research questions that require us to understand the complexity of people’s social and cultural lives.

**Approach:**

I draw on an ethnographic study to describe the complexities of studying maternal health in a rural area in India. I then show how the lessons learnt in that setting and context can be applied to studies done in very different settings.

**Results:**

I show how ethnographic research depends for rigour on a theoretical framework for sample selection; why immersion in the community under study, and rapport building with research participants, is important to ensure rich and meaningful data; and how flexible approaches to data collection lead to the gradual emergence of an analysis based on intense cross-referencing with community views and thus a conclusion that explains the similarities and differences observed.

**Conclusion:**

When using ethnographic research design it can be difficult to specify in advance the exact details of the study design. Researchers can encounter issues in the field that require them to change what they planned on doing. In rigorous ethnographic studies, the researcher in the field is the research instrument and needs to be well trained in the method.

**Implication:**

Ethnographic research is challenging, but nevertheless provides a rewarding way of researching complex health problems that require an understanding of the social and cultural determinants of health.

## Introduction

Social scientists working in public health see the social and cultural contexts of research participants as being of utmost importance for human health and well-being. Understanding the complexities of the social determinants of health and the context in which particular behaviours occur can help us analyse and untangle people’s notions of health and illness, which influence their health-seeking behaviour and determine more often than not the likely success of public health interventions to improve health. This paper demonstrates that ethnographic research design is a difficult but rigorous approach to researching questions that require understanding the complex social and cultural contexts of people’s lives. It is in analysing context-dependent and often intractable or complex health problems, such as why people persist in lifestyle practices despite knowledge of the risks to their health, that ethnographic methods can make their unique contribution to public health research.

In this paper, I describe the way in which ethnographic research was applied in a carefully designed anthropological study in India that explored maternal and child health care practices in the context of initiatives to reduce maternal and infant mortality. I then show how the lessons learnt in that setting can also usefully be applied in a maternity setting in the developed world, in the context of investigating another complex health question: why South Asian women seem unable to act on health messages for managing gestational diabetes mellitus, a condition they experience with alarming prevalence and which has serious impacts for their own health and that of their infants.

### Maternal mortality in less industrialised countries: the context for the ethnographic study

The Safe Motherhood Initiative in 1987 [1] was a response to a growing recognition that primary health care programs in many developing countries were unable to address the tragedy of high maternal mortality [2]. Initiatives such as the 1994 International Conference on Population and Development [2] and the Millennium Development Goals [MDG] [3] were launched to reduce maternal and infant mortality in the developing world. However, in many priority countries where 98% of maternal and child deaths occur, mortality ratios remain high. Currently, the global maternal mortality ratio is 400 maternal deaths per 100,000 live births as compared to 430 in 1990 – an annual decrease of less than 1% [4,5]. A primary aim of the various initiatives was to strive for skilled birth attendants, at least four antenatal care visits, and family planning programs. Unfortunately the uptake of these services when implemented has been very slow [5]. The questions that need to be answered are: What are the underlying reasons? What services do communities and people themselves see as needed, and how are these services best provided? These questions are too complex to be answered by a highly structured interview or survey. In the socially disadvantaged communities where the high mortality rates occur, literacy is often poor and researchers may be viewed with distrust. On the other hand, ethnographic research is well suited to finding out in a sensitive way what is influencing people’s choices, and thus what might be done to provide acceptable and improved health care services. Ethnography has advantages, but it also poses difficulties and challenges.

### Doing ethnography in India

In the early to mid 1990s, there was increasing concern about high maternal mortality in rural areas of India. In the mid-1990s, I conducted a questionnaire-based study in rural West Bengal, India, and established that the problem was greater than lack of access to health services. A small number of interviews with women suggested that socioeconomic and cultural factors were also relevant. My second study was therefore a more extensive analysis of the socioeconomic and cultural factors affecting the uptake of health care and maternal mortality in this region. A comprehensive literature review showed that, although determinants such as health and reproductive status, access to health services and individual behaviour, were largely responsible for maternal health outcomes, there were other significant known and unknown factors at play that could impinge upon maternal and child health.

At that time, the main conclusion in the literature was well represented in the work of McCarthy and Maine (1992) who analysed the determinants of maternal mortality and morbidity [6]. I adapted the McCarthy and Maine framework, which was based on the bio-medical and social determinants paradigm. They contended that socioeconomic and cultural factors were at the greatest distance from maternal disability and death; and that the health status of the mother, her reproductive status and access to health services directly influenced her pregnancy outcome. As an anthropologist, I could not agree. A woman’s health status certainly depended on access to services but what about the impact of her standing in the household and in the wider community and society, her access to and knowledge of available resources, her decision-making relating to her own health, gender, other socioeconomic resources and nutrition [5]? In addition, I also looked into the theory of ritual pollution and its relation to labour, delivery and childbirth - a powerful and enduring social control mechanism in Indian society. Concepts of purity, pollution, and defilement are a reflection of cultural attitudes to the body and the organisation of these attitudes into a status hierarchy [7].

To investigate this, I decided to conduct fieldwork in the State of West Bengal.

### Rationale for selecting study sites and sample

I speak the language of the State fluently and I had conducted previous research in one of the districts where I had built good rapport with the primary health care sector, community leaders and, most importantly the local community. I selected three more districts to give me four sites that differed in terms of key social variables that the anthropological literature identified as likely to shape birthing choices: maternal and child health care practices were different; with different maternal and child health indicators; with different socioeconomic and cultural backgrounds; and with ethnic differences in religion, caste, and tribal composition, from the east, west, north and south of the state. Together these different settings would give me data about decision making in different contexts and that would allow me to identify the critical determinants of practice.

My overall aim was to gain a deeper understanding of women’s experiences relating to their reproductive health and health seeking behaviour and to explore the influences on their and their children’s health. Data collection processes focused on observation of the daily life of the community, in-depth interviews, and focus groups. I started with key informants who would be best able to tell me what happens in their communities. Based on my literature review, key informants included primary level health care providers and traditional birth attendants who attended home births. I planned to conduct focus groups with mothers-in-law, or elderly aunts and community leaders who could also provide insights into changing care practices.

Central to the study were women who had had at least one live birth, or experienced a pregnancy ending in neonatal loss, abortion, or stillbirth, or were pregnant at the time of my study. I wanted to know about their reproductive health from the time of their marriage until they had completed their desired family size and composition, or were still trying to achieve it. A supplementary questionnaire for heads of the households (father-in-law, older brother-in-law, or the husband) sought data on socio-demographic and economic characteristics of the household and sought to find out how decisions were made relating to reproductive health issues like family planning, utilisation of health care services, including both western and traditional medicines.

To understand women’s or their family members’ reluctance or willingness to use available health services, it was important to observe the functioning of the health care centre in each study site and to observe health care providers interacting with the population they were serving to gain further insight into what influenced preference for one service over another.

I decided that spending four months at each study site would provide me with sufficient time to be part of the community under study and to explore and understand its culture and its intersection with health, well-being, and illness. If this time-period proved insufficient, I could extend my stay, as being flexible in approach around the time needed to collect data is an important feature of ethnographic research.

I arrived in the capital city of the State with great enthusiasm and here I made contact with my local mentor, piloted my questionnaire and arranged to travel to the study sites. I employed three research assistants to help me with administering the questionnaire.

### **Study site A:**

Study site A is about 184 km from the capital city, but the journey to reach this site involves first taking a train, a bus, and then a bullock-cart, an entire day of travel. To gain a smooth entry into the field, on arrival, I was met by the Manager of the Agricultural Research Centre, with whom I had made contact through my local mentor. He introduced me to the village head and other key villagers. The village head then introduced me to their youth leader who gave me a tour of the village and introduced me to the villagers. On my first meeting with the villagers, I explained the purpose of my visit. They then asked many questions to clarify the purpose of my visit, which I did honestly and openly.

The community leaders designated a school classroom (completely empty besides a blackboard) as our abode for the four months stay. There was no electricity, no running water (a well was in the school compound), no lavatory (just a hole in the ground), and a small washroom with buckets to fill in water from the well for a bath/shower. It was already winter and bitingly cold. We unrolled our sleeping bags on the floor and this was our home for the next four months. Initially, our meals were organised from a small teashop that catered to the long-distance bus drivers and truck drivers and other passers-by who stopped at this village bringing in supplies from the nearest town.

My research assistants found this setting too hard and after 10 days, I arranged for their return journey to the capital city. It proved much easier to build rapport with the community on my own. The villagers were reluctant to talk about their intimate personal experiences with the research assistants who came from the same State. Although my mother tongue, cultural and ethnic background is Bengali and I was considered of Bengali origin, people were aware that my life experiences were very different from theirs as I grew up and lived in a different state. I was considered enough of an insider to be trusted but also seen as an outsider who did not have an intrinsic network of contacts and insider knowledge about their community that could put them at any risk or disadvantage when they shared their intimate life experiences with me.

### Immersion and rapport building

Once people accepted me in their midst and became used to seeing me as part of their daily life, I started talking to women about their life, their daily activities, their concerns about their children, family, and so on. I would normally visit women’s homes and try to assist them with their daily chores and activities. They were tolerant and taught me how to milk, make puffed rice and other items. When a level of comfort was established (this became clear when women started confiding in me) I focused on gathering my data.

Administering a face-to-face questionnaire with the head of the household also broke down barriers. They questioned me about why I wanted to study their health care practices and how would they benefit from the study and why should they spend their time with me. I honestly told them that they would not benefit directly, but that future generations might benefit from the findings of the study. They were also curious about my marital status, what my husband thought about my study and why he did not accompany me, wasn’t he concerned about my safety and welfare in a strange place, etc. Being open and truthful worked wonders and they took good care of me, arranging for my meals to come from the local community leader’s home (as I was on my own), ensuring that someone accompanied me at night when I was collecting data, and that I was not alone in my temporary home, especially at night.

Before talking to women about their reproductive health, I reiterated the purpose of my trip, and sought their permission to take notes or record our conversation. The women and/or their head of household were apprehensive about signing a paper consent form, but were more than happy to talk to me. They gave verbal consent to interviews and permission to take notes of our conversations. Being willing to talk with me on tape or being happy for me to take notes, was taken as an indication of consent in these circumstances.

My data collection progressed smoothly and people were very generous in sharing their stories. I dropped in at women’s homes without an appointment (this is the way people interact), talking with them while they were busy with their daily chores. In-depth interviews took two to three visits to complete, as women talked about other more pressing issues. Some of the women preferred talking to me in the evenings after dinner at my temporary home, as they wanted some time off from their daily lives. Focus groups with women from different generations and their partners were planned, but had to be abandoned after two attempts, as they proved too chaotic and difficult with lots of arguments about what practices were good and beneficial, and how the younger generation were misled. However, this need to be flexible in my method, possible within an ethnographic approach, meant instead that I used case studies, in-depth interviews and observation to collect a range of information on health care practices. In this instance flexibility of method ensured rigour to be achieved because it enabled collection of the most appropriate and rich data to address the research questions.

### **Study site B:**

Of the different study sites, the one that offered most challenges was study site B. This study site is largely populated by tribal people and is about 300 kilometres from the capital city. Strangers are not readily welcomed in the community and my temporary home was at the primary health care doctor’s quarter. Fieldwork was painstakingly slow and frustrating, taking longer than the stipulated four months. Building some sort of a rapport with the community took almost two months, but even then I was not totally accepted and people were not as welcoming and open as they had been in study site A. They particularly distrusted anyone associated with the health care sector especially if they were family planning workers. During the ‘Emergency Period’ (25 June 1975 – 21 March 1977) in India, many of the rural poor and illiterate were forcibly sterilised (vasectomy and tubectomy) to control population growth. Even decades later, the community continued to fear that if they talked to me about their reproductive health care practices and their intimate personal experiences, I would somehow harm them. It was a difficult, time-consuming task to convince them that I was not a health care practitioner or a government official and that I was a researcher who meant no harm, but who wanted to learn about and understand their experiences so that their stories could be told.

The importance of this very different site was that it gave a powerful insight into the reason for local beliefs that determine health care decision-making. This site was deliberately chosen to ensure that diverse population groups from diverse locations were represented in the sample to provide answers to complex questions and to understand how this affected and influenced their reproductive health. A well-designed qualitative study will generally acknowledge and explore the divergence of experiences. This is often achieved through sampling and selecting for diversity.

Despite growing trust many women initially refused to talk, and then would just nod their heads and not say anything when the tape-recorder was switched on. I therefore decided to talk to the men first, early in the morning before men and women went to work (as most women in this village worked as labourers) and while women were busy with other household chores. Although I did not gain their complete trust, they were at least willing to talk to me, and encouraged their womenfolk to talk to me. They did not want to tape their interviews, but agreed that I could take notes. I had to display all my interviewing skills and coaxing powers to get them to talk about their experiences. Women felt comfortable talking when other women from their family were present, or when the traditional birth attendant was present. So one-on-one in-depth interviews ended up being group interviews. Nonetheless, these interviews provided rich data about inter-generational practices relating to reproductive health, and I was able to observe the keen interaction between the women about their different experiences. I had to schedule interviews either late in the evenings or very early in the mornings, as very few remained in the village during the day, with the exception of elderly women and children. I utilised this time to complete my interview notes, and review them. I had plenty of time to talk to the health care providers and observe the functioning of the health centre.

### **Study site C:**

Site C was predominantly Muslim and resembled a small satellite town full of people, predominantly men. This site was specifically chosen to explore the influence of religion on women’s reproductive choice and health, and how it affected child health. This study site boasted of modern amenities and facilities such as: small stationery stores, chemist, grocers, western medicine clinics (having ultrasound facilities), homeopathic dispensaries, snack bars, cloth merchants, hardware store, bank, police station, post office, railway station, and video parlours. I was housed at a primary health care doctor’s residence as he was on leave. The villagers were very welcoming and forthcoming and everyone wanted to talk to me.

### **Study site D:**

Site D was approximately 178 kilometres from the capital city and easily accessible by rail and road, and had all the modern amenities and facilities at its doorstep. This site had a wide range of people from different religious, cultural and caste backgrounds and most of them worked in professional jobs. This site was ‘developed’ in terms of socioeconomic and geographic determinants and was chosen to understand if the impacts for women’s reproductive health and their children’s health were different or similar to that of other study sites.

## Data analysis

Data analysis was completed when I returned from the field. Analysis of in-depth interviews and case studies was conducted manually by reading and re-reading transcripts, which enabled identification of emerging analytical categories. Analytical categories were identified by repetition and classification of codes, each building on the one preceding, to achieve results successively. These were then organised based on emerging themes and sub-themes, exploring commonalities in women’s experiences, but also taking into account any divergent experiences. Transcripts were coded and analysed thematically to retain access to the respondent's own categories [8,9]. As new categories emerged, previously coded data were re-coded and re-organised. Themes and sub-themes were crosschecked across the transcripts for consensus in order to emphasise the meanings of the social situation to participants, and to connect the social situation and the cultural patterns within it.

What I saw in my data was the way in which gender, patriarchy, culture, norms, mores, and tradition, dictated, moulded, and guided women’s role and status within the household and society. Women understood and followed prescribed roles both at the household and societal level, and these behavioural patterns affected their reproductive careers, which, in turn, influenced their health seeking behaviour during their reproductive years [10].

Across all four sites, I could show that health services provided by the primary health care sector had had limited impact. For example, only around a quarter of the surveyed population sought treatment from their primary health care services for major or minor illnesses. Although services were available and accessible within a five-kilometre radius women were cautious in utilising these services, especially at labour and delivery (nearly two-thirds of the women in the study had opted for home birth assisted by a traditional birth attendant or family/friend). Women from study site B were apprehensive and cautious in using reproductive health services because of the past experiences of their family members and community members during the Emergency Period in India [10]. Even during prolonged labour, and/or complication during labour and birth, 97% of the women from study site B chose to depend entirely on their traditional birth attendant, and did not seek assistance from their primary health care sector. This demonstrates the degree and level of suspicion, and distrust that still existed with the reproductive health care services after almost two and half decades since the Emergency Period and the time of my field work. But, on the other hand, women from study site C used the available modern diagnostic technology to terminate unwanted female foetuses [11].

Medical pluralism flourished in the study areas (people used more than one medical system simultaneously at all study sites). Health seeking behaviour was flexible, and people switched from one medical system to another depending upon cost, time, perceived effectiveness, and viability. The strong influence of culture and tradition reigned especially, for seeking treatment for infants and very young children, practices related to pregnancy, delivery, childbirth, postpartum, and lactation. Socioeconomic factors such as housing, crowding, lack of plumbing, quality of drinking water, source of fuel, lighting, poverty, undernourishment, malnourishment, and low income, low levels of education and literacy of the mother and the family, religion, caste, tribal affiliation all affected the health status of a family, and in particular, that of the mother, which consequently affected her child’s health. My conclusion was that maternal morbidity and mortality rates would only improve if these factors were addressed and not only the provision of good primary health care [10]. In addition, the way in which services are introduced should be appropriate for the community so that they feel comfortable in utilising them.

## The lessons learnt

The most important contribution of ethnographic research is that it encourages us to be open-minded about possibilities in deciding which framework/theory will guide the research and which methods will elicit the best data for a particular phenomenon under study. It promotes new ways of thinking about and uncovering the cultural frameworks, in analysing their structure and content and using this as a basis for explaining what is going on. It provides insights into the meaning of social and cultural perspectives and the possibilities for change. It can provide detailed, in-depth description of everyday life and practice of people under study, and explain how these influence behaviour and practice [12].

How then can the lesson learnt doing an ethnographic study in India, be applied elsewhere? It is important to begin by acknowledging that ethnographic method, and its adaptation in in-depth interview studies is time-consuming and demanding, but it has real value when more structured research methods fail to deliver the information required. For example, when the aim is to understand persistent public health problems such as low compliance with long term medication for chronic disease; or apparent incapacity for lifestyle change despite knowledge about the risk of developing a chronic disease, and so on, ethnographic methods have much to offer. In these examples, interventions have often been focused primarily on changing individual behaviour and on developing educational programs to bring about change, and they have very often met with a disturbing lack of success.

What is often needed in these situations is a greater understanding of the social and cultural context of decision-making. For example, if lifestyle behaviour proves difficult to change there is a need to investigate why this is so, including consideration of the ways in which aspects of the social and cultural context may constrain an individual's capacity to modify behaviours.

One example from an ongoing program of research illustrates this, drawing on an investigation of a study of South Asian women’s experiences of living with Gestational Diabetes Mellitus (GDM) in Australia [13]. I consider the different stages of the study as a means of identifying the value of an ethnographic approach in disentangling the complexities of how women grappled with their diagnosis of GDM, and consequently for understanding why the standard health messages they had received were not having the desired impact.

### Establishing the state of knowledge of the problem: the literature review

An extensive literature search revealed no studies had been undertaken of South Asian women’s experiences of living with GDM although it was acknowledged in the literature that they are a group at significantly increased risk [14-16] so an investigation of South Asian women and GDM clearly had a role in filling this gap in knowledge. It seemed to me that the lack of knowledge about how South Asian women responded to the diagnosis and what actions they took to manage their condition pointed to the need for an exploratory, qualitative study where an ethnographic approach would be advantageous. Understanding the experiences of this high risk group of immigrant women would require sensitive exploration. An ethnographic approach would elicit the meanings of the social and cultural perspectives in a sensitive and empathetic manner. The insights to be gained from a sensitive ethnographic exploration of women's experiences of the condition and their care would surely assist health care providers’ in better supporting women to manage and cope with their condition in order to avoid any adverse pregnancy outcome; and in the long-term to reduce the risk of manifestation of type 2 diabetes – a major public health concern in Australia, as it is elsewhere.

### The theoretical framework

Social scientists argue that all knowledge is theory-laden [17-20] and that methods are theory-driven [21-24]. In this particular case, we sought to investigate:

• women’s coping and management strategies; their attitudes and behaviour; the impact of the diagnosis on the woman, partner and family, and the support available;

• how women understood and implemented the advice and management strategies provided by their health care providers

• women’s understanding of GDM and diabetes after birth; and

• measures women used to improve pregnancy outcome, and their prevention strategies to delay/prevent onset of type 2 diabetes in the long-term.

To address these questions, health belief and ecological models, within a social determinants framework, were adapted to identify and understand the factors affecting management of GDM for women. Further, we explored if cultural practices, class or community-related practices were strongly influencing women's behaviour; and how would behaviour modifications that women were advised to make, interact with their ethnic and cultural identities? Were there community practices or conventions that needed to be understood? These theoretical ideas provided an initial basis for sample selection to inform the areas to be investigated in the study.

### **Immersion and rapport building**

The need for immersion and rapport building in the field is a step that is all too often overlooked. From my experiences in India I knew that researchers need to be sufficiently integrated into the community being studied to be able to gain people's trust. This is true in the maternity care setting, just as it was in my study in rural India. Rapport building takes time and perhaps a special kind of person, a good empathetic listener, and someone who does not interrupt or interfere. While the GDM study did not require the same degree of immersion and rapport building as in my earlier study, it was important that I took the time to get to know the staff in the hospital GDM clinic, explaining the study's purpose and learning about how care was provided and some of the challenges the staff faced. In addition, I needed to spend time with the women attending the clinic, also explaining my interest in doing the research, explaining what participation in the research would involve, and obtaining written consent. This study focussed on understanding the barriers women faced in successfully managing their diabetes in pregnancy and exploring their lived experiences of GDM. Hence, it was appropriate to use face-to-face in-depth interviews to gather information.

### **Sampling and recruitment**

Rigorous method, including ethical conduct, is central to all research.

I knew that our potential research participants were scattered across a number of different geographical settings; came from different socioeconomic stratum, spoke a variety of languages; could be recent migrants or second-generation immigrants. Who, then is best placed to conduct this research?

### Ethnographic method

Well designed and rigorous ethnographic research is dependent upon the skills of the researcher. The researcher needs to be a skilled and empathetic listener with a lot of patience, integrity and honesty, and someone who can engage with people of diverse backgrounds. The researcher needs to have skills in building rapport with the research participants and to be intimately involved in observing, interviewing, probing and then analysing and interpreting the data collected. This therefore, is no task for an inexperienced researcher nor can it be done by a research assistant. In ethnographic research, the researcher is the research instrument and needs to be well trained in the subtleties of the method.

As evidenced from my earlier study, I had to ensure that the sampling reflected diversity with respect to the key social determinants identified in my review of the literature: diverse socioeconomic backgrounds, different countries of origin in South Asia, different religions, and varied proficiency in English, to name the most important factors. The study design also needed adequate flexibility around timing and approach, depending on what I found once my interviews began. Given the need for immersion and rapport building, I chose one particular site, a tertiary level maternity hospital in Melbourne, Australia, with a GDM clinic attended by many South Asian women. I concentrated on collecting information through one-on-one in-depth interviews with women, and also their partners, as it was soon discovered partners often accompanied women and women wanted them involved. The theoretical framework helped me focus the study on the social and cultural issues likely to be important and guided the analysis of data obtained from my observations and from in-depth interviews, both formal and informal with the study participants.

### **Data collection and analysis**

Data were collected in tape recordings but these were also supplemented with memo notes on observations in the clinic and sitting in on a GDM education session at the hospital. Analysis was a complex process involving large amounts of textual data; therefore the process of data analysis was explicit at every step of the process [25,26]. In-depth interviews were analysed by listening to the tape recordings after each interview session was concluded and cross-checking with the field notes and observational notes. Once the tape recordings were transcribed, multiple reading of the transcripts facilitated identification of commonalities and differences in experiences. I found that aspects of culture, norms and traditions all affected women’s lifestyle in pregnancy and shaped their behaviour. For example, socioeconomically disadvantaged women in the Indian sub-continent experience adverse pregnancy outcomes because of under-nutrition and/or malnutrition and due to strenuous physical labour [27]. Therefore, expectant mothers are provided more food and advised rest to avoid any adverse pregnancy outcome [13]. These allowed explanation of why South Asian women behaved in the way they did during pregnancy [13].

Findings provided in participants own words and phrases with an explanatory discussion gives insight into the linkage with extant theories or contributes new theoretical ideas and how it is related to other work [28]. As ethnographic researchers we have to convince our audiences that adequate time has been spent observing all relevant activities to be able to interpret and draw robust conclusions about normal and unusual kinds of behaviour. For example, are we able to talk conclusively about the lifestyle practices women followed in pregnancy and whether they were good or harmful for the mother and the baby? Such topics require sensitive discussion and explanation, taking into account the specifics of women’s context, and cultural norms and meanings.

The above-mentioned study facilitated in deepening our understating of why South Asian women diagnosed with GDM were not convinced and/or were unable to implement the advice given by health care providers relating to diet and exercise in pregnancy was possible because an ethnographic approach was used in gathering information. This deeper understanding of women’s experiences may not have been easily garnered by other more traditional research methods.

This study has further led me to develop the next phase, which will apply similar methods to further our understanding about health care providers’ perspectives for caring for South Asian women with GDM in Australia.

### Conclusion and implications

One of the challenges faced when conducting an ethnographic study is that it evolves, depending on what is found in early data collection. Clearly, it is not possible to describe all aspects of the approach to be taken at the planning stage. However, experienced researchers should be able to indicate the range of what they expect to find in the field. Designing a research study of this kind requires knowledge of both the public health and the theoretical literatures in order to focus data collection on the best sites, most relevant to the complex health problem to be investigated.

Conducting the study requires a high level of research skill. Working in the field can be lonely and challenging as researchers work to develop rapport and build relationships in order to learn about people’s experiences. The need is for an empathetic listener, yet one who is able to retain sufficient distance to be able to analyse the contextual and personal data in an impartial way, in order to provide good evidence for health policy and practice. The reward for the researcher is being able to engage with, listen and learn first-hand about research participants' concerns. The value for advancing public health knowledge is the deeper understanding that arises from using ethnographic methods to investigate the complexities inherent in the social determinants of health, whether in developing or industrialised nations. One of the most important contributions of ethnographic research lies in its unique contribution in understanding and unravelling the complexities, meanings and underlying raison d'être, embedded in people’s everyday lives and in their lived experiences, informing practice and policy.

## Competing interests

The author declares that she has no competing interests.

## Authors' contributions

MB wrote and read the final manuscript.
